# A Comparative Study on the Hemodynamic Performance Within Cross and Non-cross Stent-Grafts for Abdominal Aortic Aneurysms With an Angulated Neck

**DOI:** 10.3389/fphys.2021.795085

**Published:** 2021-12-02

**Authors:** Ming Qing, Yue Qiu, Jiarong Wang, Tinghui Zheng, Ding Yuan

**Affiliations:** ^1^Department of Applied Mechanics, Sichuan University, Chengdu, China; ^2^Yibin Institute of Industrial Technology/Sichuan University Yibin Park, Yibin, China; ^3^West China Biomedical Big Data Center, West China Hospital, Sichuan University, Chengdu, China; ^4^Department of Vascular Surgery, West China Hospital, Sichuan University, Chengdu, China; ^5^Med-X Center for Informatics, Sichuan University, Chengdu, China

**Keywords:** abdominal aortic aneurysm, endovascular aneurysm repair, cross limb technology, stent-graft, hemodynamics

## Abstract

**Objectives:** Cross-limb stent grafts for endovascular aneurysm repair (EVAR) are often employed for abdominal aortic aneurysms (AAAs) with significant aortic neck angulation. Neck angulation may be coronal or sagittal; however, previous hemodynamic studies of cross-limb EVAR stent grafts (SGs) primarily utilized simplified planar neck geometries. This study examined the differences in flow patterns and hemodynamic parameters between crossed and non-crossed limb SGs at different spatial neck angulations.

**Methods:** Ideal models consisting of 13 cross and 13 non-cross limbs were established, with coronal and sagittal angles ranging from 0 to 90°. Computational fluid dynamics (CFD) was used to capture the hemodynamic information, and the differences were compared.

**Results:** With regards to the pressure drop index, the maximum difference caused by the configuration and angular direction was 4.6 and 8.0%, respectively, but the difference resulting from the change in aneurysm neck angle can reach 27.1%. With regards to the SAR-TAWSS index, the maximum difference caused by the configuration and angular direction was 7.8 and 9.8%, respectively, but the difference resulting from the change in aneurysm neck angle can reach 26.7%. In addition, when the aneurysm neck angle is lower than 45°, the configuration and angular direction significantly influence the OSI and helical flow intensity index. However, when the aneurysm neck angle is greater than 45°, the hemodynamic differences of each model at the same aneurysm neck angle are reduced.

**Conclusion:** The main factor affecting the hemodynamic index was the angle of the aneurysm neck, while the configuration and angular direction had little effect on the hemodynamics. Furthermore, when the aneurysm neck was greatly angulated, the cross-limb technique did not increase the risk of thrombosis.

## Introduction

Endovascular aortic aneurysm repair (EVAR) is the primary alternative for treating an abdominal aortic aneurysm (AAA). When an AAA has unfavorable anatomy, such as a significant aneurysm neck angulation or widely splayed common iliac arteries, conventional EVAR, which employs contralateral (non-cross) iliac limb deployment, is difficult and time-consuming to cannulate, and it is also followed by poor outcomes (Sternbergh et al., 2002; Rodel et al., 2009). Focusing on the AAAs with severe anatomy, Ramaiah et al. first proposed and applied cross-limb EVAR to seven patients in which the bifurcated stent grafts (SGs) were twisted into a cross-limb configuration. Their post-operative follow-ups indicated that this cross-limb technique could make the stent smoother and achieve better short and medium-term effects (Ramaiah et al., 2002; Yagihashi et al., 2017). However, its long-term prognosis is still questionable (Sternbergh et al., 2002; Dattani et al., 2015).

Experimental and clinical evidence indicates the important role of flow dynamics within the stent grafts in the occurrence of post-EVAR complications, including stent migration and thrombosis (Raptis et al., 2017a). Therefore, comparing the safety and efficacy of cross- and non-cross-limb stent configurations has been studied from a hemodynamic perspective using computational fluid dynamics (CFD). Recall that cross-limb EVAR is introduced and mostly adopted in AAAs with angulated necks. Moreover, the neck angulation may be coronal and/or sagittal; namely, the hemodynamic alteration from a conventional non-cross EVAR may be caused by different spatial azimuths. However, previous hemodynamic studies on the cross and non-cross EVAR mostly adopted idealized and realistic models without neck angulation (Georgakarakos et al., 2014; Stefanov et al., 2016; Liu et al., 2018). Moreover, although there have been comparative studies using patient-specific EVAR models in which necks may be angulated, a particular AAA has individualized geometry, and other geometric features (including the stent configuration) may cause the hemodynamic differences between the cross and non-cross SGs (Efstratios, 2012; Stefanov et al., 2016). This suggests that the hemodynamic comparison between cross and non-cross limb EVAR is still the subject of controversy. Some researchers have suggested that cross and non-cross EVAR demonstrate similar hemodynamic performance, and a cross limb configuration may even inhibit the thrombosis formation because it increases the wall shear stress (WSS) and helicity (Shek et al., 2012). Nevertheless, some studies have suggested that cross-limb configurations might result in strip areas vulnerable to thrombosis formation, potentially resulting in long-term stent graft failure (Liu et al., 2018).

The present study investigates the influence of the cross-limb technique on both near-wall and intragraft flow dynamics. In particular, the impact of neck angulation, either coronal or sagittal, on local flow features is analyzed by performing CFD simulations on idealized models of the cross and non-cross limb EVAR. Such an idealized model-based approach will enable (by varying the neck angulation while keeping other geometrical factors constant) the identification of whether and to what extent a cross-limb EVAR results in the hemodynamic alteration from a conventional limb technique. These results may or may not encourage its use to improve the clinical outcome of EVAR.

## Methodology

### Models and Grids

**FIGURE 1:**
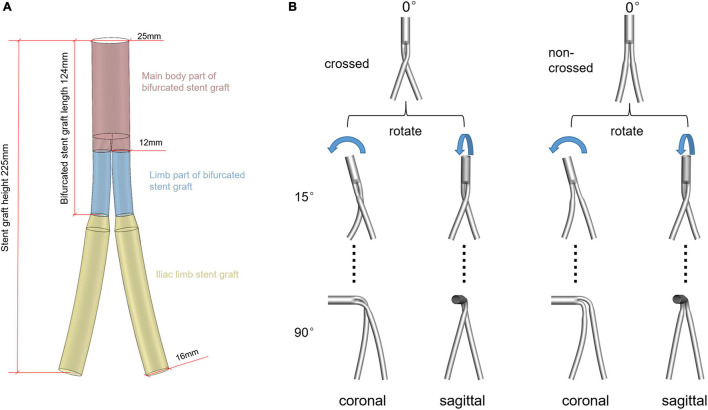
**(A)** Schematic diagram of the 0° model. Different colors represent different parts of the SG system. **(B)** Schematic diagram of the establishment of ideal models, rotating the aneurysm neck in plane and space position under the control of the overall model height unchanged. The first line is the model schematic diagram of the aneurysm neck without angulation. The second and third lines represent the model schematic diagram with an angle of 15° and 90° after the rotation of the aneurysm neck, respectively. The rest of the aneurysmal neck rotated 30°, 45°, 60°, and 75°, respectively, with 26 models.

The model is based on the Endurant TM abdominal aortic stent (Medtronic^®^, Inc., United States). The model is established under the guidance of clinicians, and the model size is selected through the given SG size parameters in device instructions for use (IFU). It is divided into three parts: the main body part of the bifurcated SG, the limb part of the bifurcated SG, and the iliac limb SG. The inlet and outlet diameters of the bifurcated SG are 25 and 12 mm, respectively, and the diameter of iliac limb SG is 16 mm. The limb and the overall length of the bifurcated SG is 45 and 124 mm, respectively. For a fair comparison, the height of the entire stent graft system was maintained at 225 mm (Figure 1A).

Two series of idealized cross and non-cross limb SG models were generated for this study, in which the necks were either angulated in the coronal or sagittal plane (Figure 1B). Although Medtronic’s instruction manual stipulates that the applicable maximum angle of the aneurysm neck is 60°, the SG is often used for aneurysms with a neck angle larger than 60° in clinical practice (Herman et al., 2018). Moreover, the maximum neck angle after the stent deployment does not exceed 90°. Therefore, in the current study, the neck angles ranged from 0–90° with a spacing of 15°.

**FIGURE 2:**
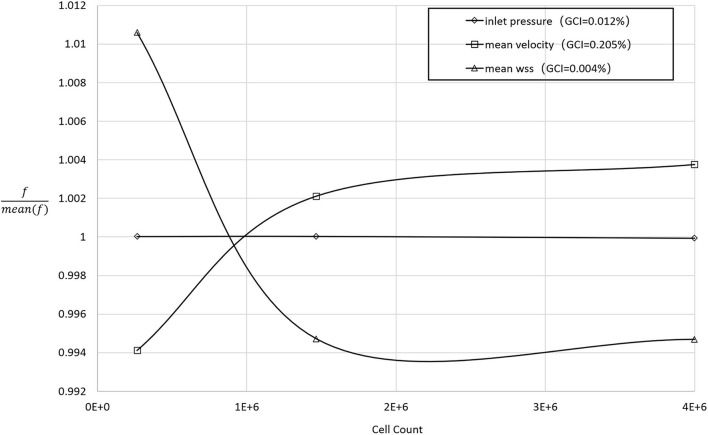
The normalized grid convergence of mean WSS, mean velocity, and inlet pressure plotted against the number of mesh cells in the model domain. The results of the GCI calculations are included in the legend.

ANSYS ICEM (ANSYS, Inc., Canonsburg, Pennsylvania, United States) was adopted for the grid generation. The meshes consisted of a mixture of unstructured tetrahedral and structured hexahedral elements. To improve the computational accuracy of the near-wall hemodynamic parameters, viscous boundary layers (height = 1, ratio = 1.2, numbers = 7) were imposed adjacent to the vessel walls of each model. Finally, the meshes for each configuration contained between 1.4 and 2 million nodes. To determine a sufficient (uniform) mesh refinement level, we applied the grid convergence index (GCI) to the steady-state simulation using the contraction peak condition. Figure 2 shows the grid convergence of the inlet pressure, the average WSS of the SG, and the average velocity. The GCI of all test variables was less than 2% (Kelsey et al., 2016), which is considered a small enough spatial discretization error to refine the grid further.

### Governing Equations and Boundary Conditions

The previous study on the hemodynamics within EVAR stent grafts generally assumed the blood is a Newtonian fluid. However, it is reported that the blood models of non-Newtonian fluid and Newtonian fluid may lead to six to eight times difference in the near-wall hemodynamic parameter values, especially at high inlet velocity (Iasiello et al., 2017). Therefore, the current study assumed the blood to be an incompressible, non-Newtonian fluid with a fluid density of 1,050*kg*/*m*^3^. In addition, Carreau fluid model which relates approximately with the experimental data was adopted for the properties of non-Newtonian fluids (Pandey et al., 2019):


(1) $$\eta \,\left(\dot{\gamma }\right)=\eta_{\infty }+\left(\eta_{0}-\eta_{\infty }\right)\,{\left[1+{\left(\lambda \,\dot{\gamma }\right)}^{2}\right]}^{\left(n-1\right)/2}$$

where η_0_ and η_∞_ are zero shear rate viscosity and infinite shear rate viscosity, respectively, and λ is the relaxation time constant. The values are η_0_=0.056*Pa*, η_∞_=0.00345*Pa*, *n* = 0.3568, and λ=3.313*s* (Johnston et al., 2004).

**FIGURE 3:**
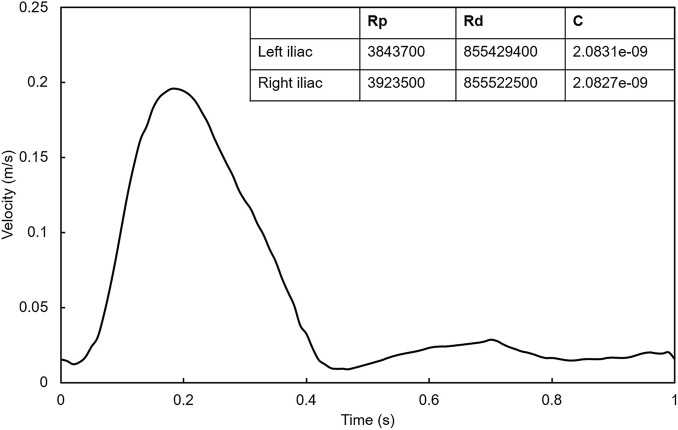
Inlet pulsating velocity waveform. The table in the figure shows the RCR parameters calculated by trial-and-error method at the outlet of left iliac and right iliac.

First, to ensure the full development of blood flow in the stent, we extend the length of the model inlet by 10 times the diameter. Second, the pulsating velocity waveform (Xiong et al., 2021) was used at the inlet of all models (Figure 3). Third, the computational models were geometrically asymmetrical, phase shift of pressure and different flow splits at two iliac outlets may occur (Grinberg and Karniadakis, 2008). Therefore, the flow split between two iliac branches in the models was regulated by the Windkessel model (Kyriakou et al., 2020), where their RCR parameters were matched for 1 million times by trial-and-error method. The matching blood pressure was the iliac artery outlet blood pressure (100/80 mmHg) given in the previous literature (Mills et al., 1970). The maximum Reynolds number of the simulated flow in the current study is 1,600, which is lower than the critical Reynolds number transferring to the turbulence flow. The numerical simulation was solved using the ANSYS FLUENT 16.0 (ANSYS, Inc., Canonsburg, Pennsylvania, United States) default independent implicit solver, and the convergence residual was 1e-4. At the seventh cycle, it is considered to achieve periodic convergence, and the results of the last cycle were used as our analysis data (Joly et al., 2019).

### Variables of Interest

Wall shear stress (WSS) and oscillatory shear index (OSI) play an important role in understanding the changes in the SG hemodynamic environment after EVAR (Nordgaard et al., 2010). Additionally, previous studies indicated that low time-averaged WSS (TAWSS) areas are prone to blood stasis, while areas with high OSI can cause platelet aggregation. Accordingly, SAR-TAWSS, the ratio of WSS area below 0.4 Pa to the entire surface area of SG, and SAR-OSI, the ratio of OSI area above 0.3 Pa to the entire surface area of SG, were introduced to quantify the hemodynamic environment on the SG (Fan et al., 2016; Xiong et al., 2020). Moreover, helical flow can reduce the concentration of platelets near the vessel wall and inhibit the interaction between platelets and the wall, and the visualization of helical structures is achieved using the local normalized helicity (LNH), average helicity (h1), and helicity intensity (h2) (Zhan et al., 2010; Shek et al., 2012; Tasso et al., 2018; Prashantha and Anish, 2019).


(2) $$L\,N\,H=\frac{\vec{\nu }\cdot \vec{\omega }}{\left|\vec{\nu }\cdot \vec{\omega }\right|}=\cos\phi$$

(3) $$h_{1}=\frac{1}{T\cdot V}\,\int_{T}\int_{V}\vec{\nu }\cdot \vec{\omega }\,dV\,dt$$

(4) $$h_{2}=\frac{1}{T\cdot V}\,\int_{T}\int_{V}\left|\vec{\nu }\cdot \vec{\omega }\right|\,dV\,dt$$

where T is the time of the cardiac cycle; ${}_{\vec{\nu }}$, velocity vector; ${}_{\vec{\omega }}$, vorticity vector; and V, the volume of the SG.

## Results

### Flow Field

**FIGURE 4:**
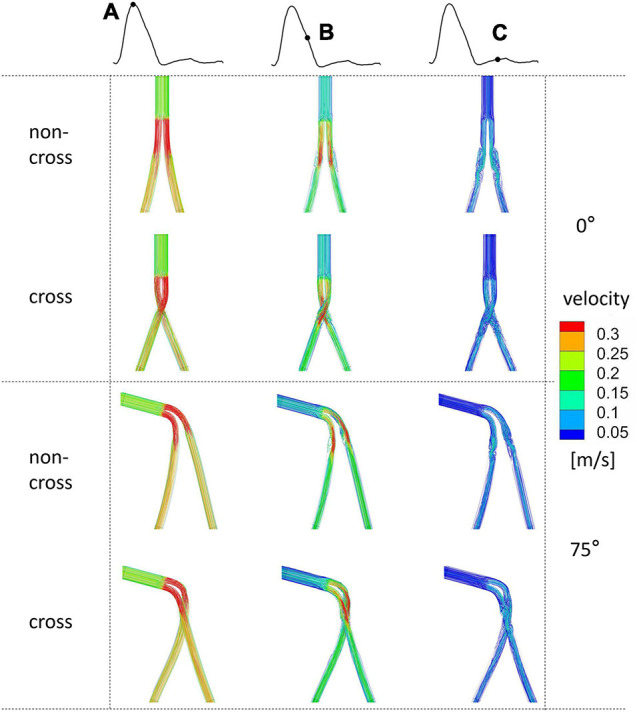
The flow chart of the systolic peak time of the ideal model with 0° and 75° coronal planes and different colors indicates different velocities. Mark **(A)** for systolic peak, **(B)** for late deceleration, and **(C)** for early diastole.

Figure 4 shows the flow chart at different times of the heart cycle. When the angle of the aneurysm neck was 0°, there was no vortex in all the SGs at the systolic peak. At late deceleration, the whirlpool structure appeared in the proximal end of the iliac limb SGs. At early diastole, the whirlpool structure develops distal to the iliac limb SGs. Compared with the stable vortex structure in the non-crossed limb, the crossed limb is more likely to form an unstable vortex structure, especially at late deceleration. With the increase in the aneurysm neck angle, there was an apparent spiral flow structure in the limb part of the bifurcated SG. The blood flow in the crossed limbs was significantly more chaotic during late deceleration and early diastole.

### Pressure Drop

**FIGURE 5:**
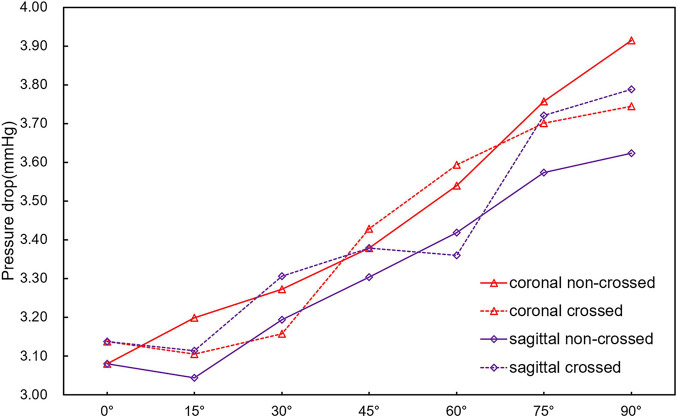
Broken line chart of inlet and outlet pressure drop at peak systolic time.

Figure 5 is a line chart of the maximum pressure drop change at the inlet and outlet of the SGs. For all the models, the pressure drop increases with the increase in the aneurysm neck angle. The change in the pressure drop in the coronal non-crossed limb SG was the most obvious, and a pressure drop in the 90° SG was 27.1% higher than that of the 0° SG. The change in the pressure drop in the sagittal non-crossed limb SG was the smallest (only increasing by 18%). At the same aneurysm neck angle, the maximum difference in pressure drop between different models was only 8.0%. The results showed that different configurations and angular directions had little effect on the pressure drop, while the aneurysm neck angle had a significant influence on the pressure drop.

### Wall Shear Stress

**FIGURE 6:**
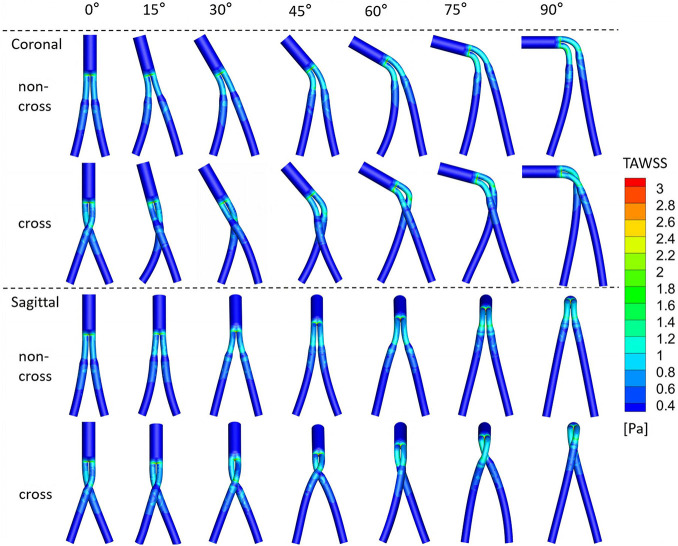
Contours of TAWSS, with different colors to represent the magnitude of TAWSS.

**FIGURE 7:**
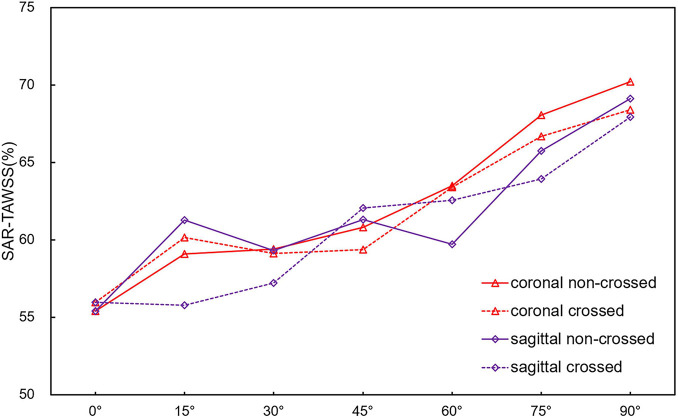
Line plot of SAR-TAWSS with aneurysm neck angle.

Figure 6 shows the TAWSS cloud picture of the model. Most areas of all models have a TAWSS less than 2 Pa. The areas larger than 2 Pa were mainly concentrated in the limb part of bifurcated SGs and proximal to the iliac limb SGs. The distal region of iliac limb SGs was smaller than 0.4 Pa. Figure 7 is a line chart of the changes in the model SAR-TAWSS (area ratio of WSS less than 0.4 Pa). The SAR-TAWSS of all models increased (from 21.7 to 26.8%) with the increase in aneurysm neck angle. Different configurations and angular directions had little effect on SAR-TAWSS, but the change in aneurysm neck angle mainly affected SAR-TAWSS.

**FIGURE 8:**
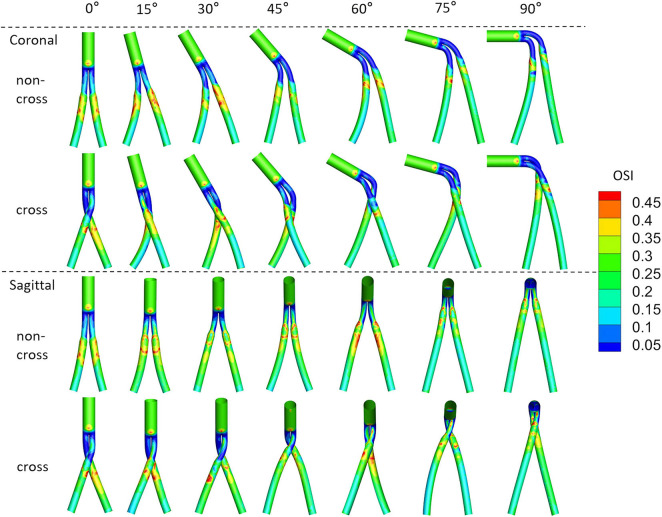
Contours of OSI, with different colors to represent the magnitude of OSI.

Figure 8 shows the OSI cloud picture of the model. The areas with very high OSI [greater than 0.3 (Xiong et al., 2020)] were concentrated in the distal end of the main body part of bifurcated SGs and the proximal end of the iliac limb SGs. The areas with very low OSI [less than 0.05 (Cilla et al., 2020)] are concentrated in the limb part of bifurcated SGs.

**FIGURE 9:**
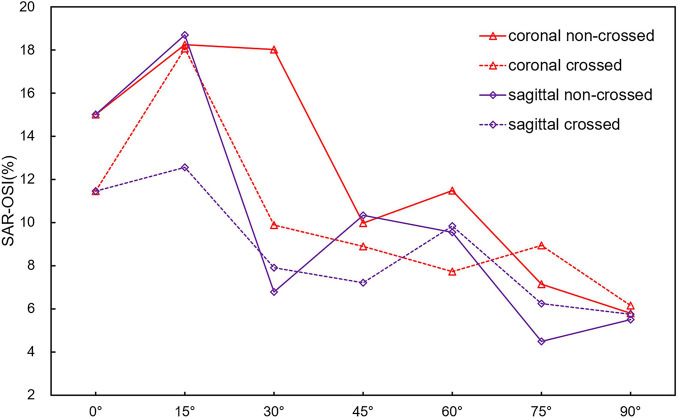
Line plot of SAR-OSI with aneurysm neck angle.

Figure 9 is a line chart of the changes in the model SAR-OSI (area ratio of OSI greater than 0.3). When the aneurysm neck angle is less than 45°, the SAR-OSI is greatly affected by the configuration and angular direction, with a maximum difference of 8.15% between crossed and non-crossed limbs and a maximum difference of 11.25% between the coronal and sagittal position. When the aneurysm neck angle was more than 45°, the difference in SAR-OSI among different models decreased gradually. At 90°, the maximum difference was only 0.65%.

### Helicity

**FIGURE 10:**
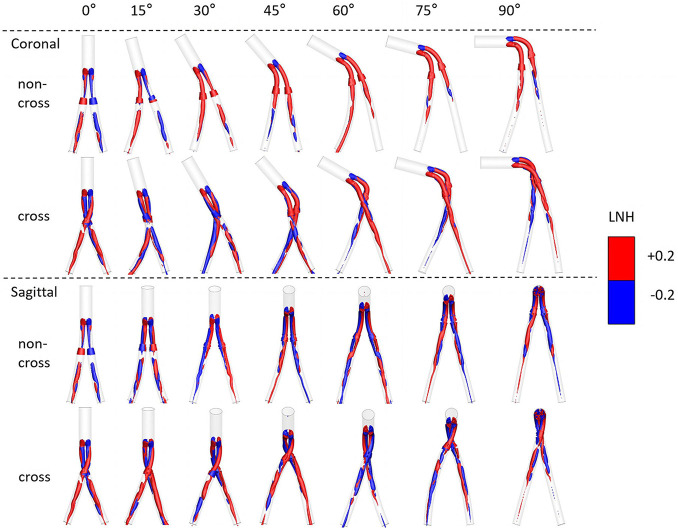
Visualization of intravascular LNH iso-surfaces.

Figure 10 shows the LNH cloud picture of the peak systolic time. In the model, left-handed and right-handed helical structures (blue and red) appear almost simultaneously. The helicity flow structure of coronal non-crossed limbs was the lowest in all models. The helicity flow of crossed limbs was significantly higher than that of non-crossed limbs. However, with the increase in the aneurysm neck angle, the difference in helical flow intensity decreases gradually.

**FIGURE 11:**
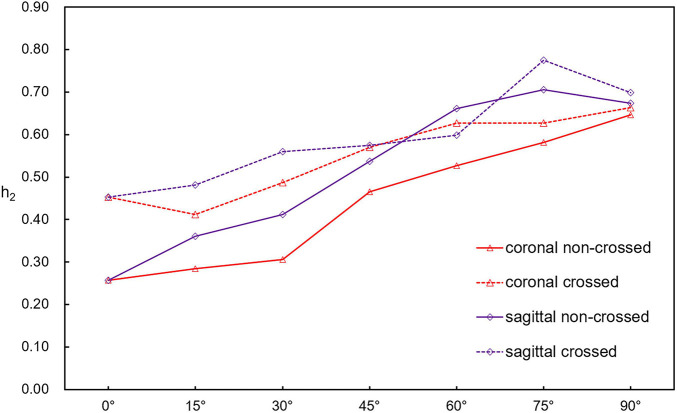
Line plot of helicity h2 with aneurysm neck angle.

Figure 11 shows the line chart of the variation of helical flow intensity (h2). The h2 of all models increased with the increase in the aneurysm neck angle. The helical flow intensity of coronal non-crossed limbs is always the smallest. When the angle of the aneurysm neck is less than 45°, the helical flow intensity of crossed limbs is higher than that of non-crossed limbs. When the angle of the aneurysm neck was more than 45°, the difference between the intensities decreased. For the same configuration, the helical flow intensity of the sagittal model is higher than that of the coronal model. The results show that when the aneurysm neck angle is small, different configurations, angular direction, and angle will affect the helical flow intensity. However, with a further increase in the aneurysm neck angle, the difference between the configuration and the angular direction decreases. At 90°, the maximum difference in helical flow intensity is only 5%.

## Discussion

The cross-limb EVAR was introduced for AAA patients with significant aneurysm neck angulation or widely splayed common iliac arteries. It significantly reduced the cannulation time compared with a conventional non-cross EVAR. However, whether or not the cross-limb stent strategy helped to decrease the incidence of post-EVAR complications (in the face of adverse AAA anatomy) is still in doubt. The current study constructed 26 cross and non-cross limb EVAR models with various coronal and sagittal angulated necks and subsequently numerically investigated the flow patterns in these models. A comparative evaluation of the hemodynamic performance was done in terms of the helical flow strength, flow field, WSS, and pressure drop.

First, this study revealed that the pressure drop of the stent increased with the increase in the AAA neck angle for both stent configurations. The blood pressure drop is one of the essential factors affecting blood flow status in the stent. If there is a deviation from the physiological state, the elevated pressure drop will result in disordered blood flow (Raptis et al., 2017a), such as that in the sac of AAA. Because the pressure drop increases significantly, there will be irregular turbulence and eddy currents will form (Qiu et al., 2018). In addition, after intravascular repair, a higher pressure drop in the stent will lead to increased loss of kinetic energy of blood flow through the stent, which in turn affects distal lower extremity vascular perfusion. This excessive pressure drop may increase cardiac output power, which eventually increases cardiac burden (Tasso et al., 2019). Although, the 90° non-crossed model has a maximum additional pressure drop of 27.1% compared with the 0° model. However, from the pressure drop value, the difference between the maximum and minimum pressure drop is only 1 mmHg. Accordingly, different SG configurations and aneurysm neck angle have little effect on the pressure drop.

Second, TAWSS is an essential hemodynamic parameter that directly acts on the endothelial layer. Low TAWSS is accompanied by unstable flow conditions such as turbulence, blood recirculation regions, and blood “stagnation” regions, resulting in an increased risk for thrombosis and intimal hyperplasia (Papaioannou and Stefanadis, 2005). The simulation results show that the proportion of the low TAWSS regions is mainly affected by the neck angle, while the configuration and angular direction have little effect on it, indicating that the use of the cross-limb technique does not increase the risk of thrombosis. Additionally, the high OSI area, which decreased with the increase in the aneurysm neck angle in a proportional manner, was mainly concentrated at the proximal end of the iliac limb SGs. High OSI area was reported to cause platelet aggregation, increase the retention time of coagulation-promoting particles and result in thrombosis (Nordgaard et al., 2010; Liu et al., 2018). Therefore, we speculate that there may be a risk of thrombosis in the proximal end of the iliac limb SGs.

Third, previous studies have demonstrated the transport effect of eddies on blood flow and the development of swirls in the cardiac cycle which brings platelets to low-speed wall reflux areas, resulting in thrombosis (Kelsey et al., 2016). From our model, it is observed that the proximal end of the iliac limb SGs will produce a vortex, and during the diastolic period, it will develop downstream and spread to the wall area, which is also consistent with the danger areas of TAWSS and OSI. The stable swirl structure formed by non-crossed limbs near the wall may increase the risk for thrombosis. Crossing limbs destroy this stable whirlpool structure, suggesting that crossing limbs do not increase the risk of thrombosis which agrees with the latest clinical report that there were no significant difference in limb occlusion between the two limb configurations at severe angular aneurysm neck (Wang et al., 2021).

Finally, the current study observed that the helicity intensity in a crossed limb SG is significantly higher than that of the non-crossed limb SG when the neck is coronal angulated, which may help to inhibit platelet adhesion and hinder the formation of acute thrombosis (Zhan et al., 2010, 2011; Shek et al., 2012; Raptis et al., 2017a). However, when the neck exhibits sagittal angulation (and if the neck angulation is greater than 45°), the helicity intensity remains similar for the two SG configurations.

The current study has presented TAWSS, OSI, and a few other parameters which are suggested to be related to the initiation and development of thrombosis. Among which, TAWSS represents the WSS magnitude, its particular values are highly related to the inlet flow rate. As a result, if the patient-specific inlet velocity is not available it is difficult to decide the threshold values of abnormal TAWSS for a particular patient. In the contrast, OSI, an indicator of the pulsatility of WSS direction, results from the flow unsteadiness over a cardiac cycle, and if the flow remains laminar, the general flow features of the flow field within the SGs are mainly decided by the geometric features of the computational models. Maybe that’s why a good match between abnormal OSI and thrombosis occurrence has been reported even though no patient-specific boundary condition was available (Cao et al., 2020). In addition, the flow pattern can be directly visualized by 4D MRI in the clinical application (Liu et al., 2014). We believe that the relation of flow pattern to vascular disease is needed to be further explored in the future CFD application.

## Limitations

There are some limitations to this study. First, the aortic model excluded the suprarenal branch arteries, and it has been reported that the inclusion of the aorta upstream of the renal artery can alter the hemodynamics of the inlet boundary fluid state (Sternbergh Iii et al., 2004). Second, this study only included Medtronic Endurant I covered stents, and the results were limited to explain the Endurant series of hemodynamic stent models. Matsagkas et al. showed that the hemodynamic indexes of different laminating stents might significantly differ from the actual model (Raptis et al., 2017b). Finally, previous studies have proposed that the folds caused by the bending of stent grafts will affect the hemodynamics near the wall (Prasad et al., 2012; Kyriakou et al., 2020). *In vivo* limb stents involve many curved parts, but the current models did not consider the impact of folds, which will be the focus of our future research.

## Conclusion

In this study, we analyzed the hemodynamic changes in SGs under different configurations, angular directions, and angles. It was concluded that the use of the cross-limbed technique does not increase the risk of thrombosis under poor neck anatomy and the main factor affecting the hemodynamic index was the angle of the aneurysm neck, which suggests that the hemodynamic changes caused by the angle of the aneurysm neck cannot be ignored when the effect of the morphology of SG on hemodynamics is investigated.

## Data Availability Statement

The original contributions presented in the study are included in the article/supplementary material, further inquiries can be directed to the corresponding author/s.

## Author Contributions

MQ: analysis and interpretation, data collection, writing the manuscript, critical revision, approval of the manuscript, agreement to be accountable, and statistical analysis. YQ: data collection, writing the manuscript, approval of the manuscript, and agreement to be accountable. JW: data collection, critical revision, and approval of the manuscript. TZ and DY: conception and design, analysis and interpretation, critical revision, approval of the manuscript, and agreement to be accountable. All authors contributed to the article and approved the submitted version.

## Conflict of Interest

The authors declare that the research was conducted in the absence of any commercial or financial relationships that could be construed as a potential conflict of interest.

## Publisher’s Note

All claims expressed in this article are solely those of the authors and do not necessarily represent those of their affiliated organizations, or those of the publisher, the editors and the reviewers. Any product that may be evaluated in this article, or claim that may be made by its manufacturer, is not guaranteed or endorsed by the publisher.

## Abbreviations

SG: Stent grafts
CFD: Computational fluid dynamics
AAA: Abdominal aortic aneurysm
EVAR: Endovascular aortic aneurysm repair
IFU: Instruction for use
LNH: Local normalized helicity
SAR-TAWSS: Surface area ratio of low time-averaged wall shear stress
SAR-OSI: Surface area ratio of high oscillation shear index
h_1_: Average helicity
h_2_: Helicity intensity.
